# The Impact of Regular Physical Activity on the Mental Health and Well-Being of Dementia Patients in High-Income Countries—A Systematic Scoping Review

**DOI:** 10.3390/geriatrics9040098

**Published:** 2024-07-29

**Authors:** Simranjeet Kaur, Sai Hyma Sree Cherukuri, Sheikh Mahbub Murshed, Adiyasuren Purev-Ochir, Erini Abdelmassih, Fahad Hanna

**Affiliations:** 1 Program of Public Health, Department of Health and Education, Torrens University Australia, Melbourne, VIC 3000, Australia; simranjit4@health.torrens.edu.au (S.K.); sai.cherukuri@health.torrens.edu.au (S.H.S.C.); sheikh.murshed@health.torrens.edu.au (S.M.M.); adiyasuren.purevochir@health.torrens.edu.au (A.P.-O.); 2 School of Pharmacy, University of Tasmania, Grosvenor St., Sandy Bay, Hobart, TAS 7001, Australia; erini.abdelmessih@utas.edu.au

**Keywords:** dementia, physical activity, mental health, well-being, falls, cognition

## Abstract

**Background:** Dementia is a term used to describe a group of symptoms impacting memory, thinking, and social abilities. Evidence suggests a positive role of physical activity in physical and mental well-being in general. This systematic scoping review aimed to gather, summarise, and analyse evidence of the impact of physical activity on the mental health and well-being of dementia patients. **Methods:** A comprehensive systematic search of mainly primary research was conducted using several databases. Peer-reviewed studies in high-income countries published between 2005 and 2023 were included. The systematic scoping review was performed using the framework outlined by the Joanna Briggs Institute (JBI) and reported using the Preferred Reporting Items for Systematic Reviews and Meta-Analyses extension for Scoping Reviews (PRISMA-ScR) statement. **Results:** Twenty-four articles (including 93,984 participants) were included in the analysis. Most articles (21) reported that physical activity (particularly regular physical activity) is associated with decreased depression score, decreased cognition impairment, and fewer falls, all of which contributed to improved well-being among dementia patients. These studies showed that there is a significant positive relationship between the level of physical activity and the level of improvement in the mental health and well-being of dementia patients. **Conclusions:** Findings from this systematic scoping review provide relatively reliable evidence that regular physical activity may improve the mental health and well-being of dementia patients. Systematic reviews and meta-analyses may be required to further confirm these findings.

## 1. Introduction

Dementia is a term used to describe a group of symptoms impacting memory, thinking and social abilities [1]. It is a syndrome that is usually of a chronic and progressive nature, and is characterised by deterioration in cognitive function beyond what might be expected from the usual consequences of biological aging [1]. Worldwide, there are approximately 55 million people with dementia, and there are approximately 10 million new cases annually [2]. In Australia, as an example, dementia has had a similar impact on the population, being ranked the second leading cause of death [3]. It is estimated that more than 400,000 Australians are living with dementia, with these numbers predicted to increase in the coming years [4]. Dementia is considered to be one of the leading causes of disease burden in Australia and is estimated to cost more than $15 billion per year [5]. It is predicted that 900,000 Australians will be living with dementia by the year 2050, thereby making dementia the third leading cause of disability-based burden for people aged 65 years or older [6]. 

Physical activity refers to any bodily movement produced by skeletal muscles and requires energy expenditure [7]. Various studies have identified the association between physical activity and a reduced risk of dementia among people aged 65 years and older [8]. Previous studies showed that physical activity protects against a number of health issues including depression [9] and cognitive issues [10,11], and reduces the risk of falls among the elderly by enhancing muscular coordination [12]. Evidence shows that older people who are more physically active tend to experience less cognitive decline [4]. Hence, it is somewhat expected that physical activity impacts the mental health and well-being of adults with dementia. However, further research is required to fully understand the specific impact of the various types of physical activity on the mental health and well-being of patients with dementia. 

This systematic scoping review focuses on understanding the impact of regular physical activity on the mental health and overall well-being of those with dementia. 

## 2. Methodology

### 2.1. Rationale for Scoping Review

A comprehensive systematic scoping review utilising the “Joanna Briggs Institute (JBI) methodology for scoping review” was performed. This search framework was first suggested by Arksey and O’Malley in 2005 [13,14]. A systematic scoping review of the literature was considered an appropriate method given the aim of this research. A scoping review approach employs a broader search strategy while also ensuring the reproducibility, transparency, and reliability of the existing knowledge in the field [15]. This review was conducted using the framework outlined by the JBI (https://jbi.global/scoping-review-network/resources# accessed on 28 September 2023) and reported using the Preferred Reporting Items for Systematic Reviews and Meta-Analyses extension for Scoping Reviews (PRISMA-ScR) statement [15]. The search strategy employed an iterative process, and was guided by the primary question: ‘What is the impact of physical activity on the mental health and well-being of dementia patients?’

### 2.2. Search Strategy

A literature search was conducted in Google Scholar, PubMed, ProQuest and Scopus to identify articles published between 2005 and 2024, using a combination of keywords and MeSH terms for physical activity, dementia, mental health, and well-being. Keywords including “physical activity”, “fitness”, “dementia”, “Alzheimer’s disease”, “elder*”, “adult*”, “improve*”, “mental health”, “improve”, “outdoor activities, “Alzheimer”, “cognitive impairment”, “cognition”, “exercise”, “activity program”, “effects”, “benefits”, “old age” and “well-being” were used and Boolean operators “OR”, “AND” were used as required. Multiple databases were chosen for this study, to improve results and reduce the risk of overlooking any eligible studies that could be used during our final appraisal [16].

### 2.3. Screening Process

Following the removal of duplicates, article titles and abstracts were screened against the inclusion criteria to determine which articles would undergo full-text review. Then, the full text of the resulting articles was reviewed for inclusion. Also, the reference list of all included articles was searched for additional articles. Articles that were considered for inclusion were: those that included participants who were diagnosed with dementia and were living in high-income countries (with no constraints on the type of dementia), peer-reviewed articles, published in the English language, and published between 2005 and 2023. Studies with any type of physical activity as an intervention, with no restriction on the type of activity, were eligible for inclusion. Exclusion criteria included articles that did not reveal any relationship or closeness to the research question or topic. We included articles that provided original data (e.g., randomised controlled trials and observational studies) as well as systematic reviews. Studies from low-income countries were excluded due to lack of research in those settings and the risk of bias in reporting due to the scarcity of data in those settings.

### 2.4. Data Extraction

Initially, data extraction was completed. The Braun and Clarke’s approach to thematic analysis was used to evaluate the data [17]. The approach consisted of six steps: 1. familiarising ourselves with the data, 2. creating initial codes for the data, 3. searching for potential themes, 4. reviewing these themes, 5. naming and defining these themes, and 6. reporting and analysing the themes [17]. Phase 6 was completed using the Preferred Reporting Items for Systematic reviews and Meta-Analyses extension for Scoping Reviews (PRISMA-ScR) guidelines [15]. Study data—including sample size, study design, recruitment and setting, data collection approach, and findings—were extracted into a template developed in Microsoft Excel, and duplicates were excluded. Findings were further summarised using an iterative coding process. These were used to develop a series of categories that broadly captured the impact of physical activity on the mental health and well-being of dementia patients.

## 3. Results

**Figure 1:**
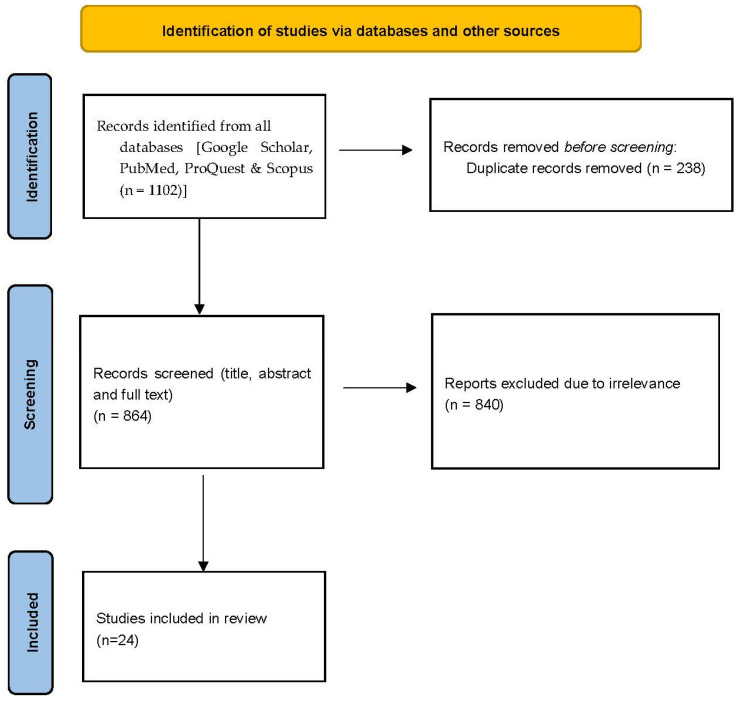
PRISMA flow chart of studies.

A total of 1102 papers were initially identified in the databases. After removing duplicates, 864 articles underwent screening based on the PRISMA screening approach. At the conclusion of the screening process, a total of 24 peer-reviewed papers met all the inclusion criteria for this review (see Figure 1).

### 3.1. Characteristics of Studies

Fifteen of the peer-reviewed publications were randomised controlled trials (RCTs). Further, one of the peer-reviewed publications was a non-randomised, non-blinded, crossover pilot study. Furthermore, three of the peer-reviewed publications were prospective observational studies. We also identified one exploratory study, one longitudinal study, and two systematic reviews (see Table 1).

**Table 1:** Characteristics of included studies.

Author, Year	Study Title	Study Design	Study Size	Intervention/Exposure	Theme	Outcome
D’Cunha et al., 2019. Australia. [18]	Psychophysiological Responses in People Living with Dementia after an Art Gallery Intervention: An Exploratory Study.	An exploratory study.	25 participants.	Art gallery outings.	Recreational activities as a form of exercise.	Improved hypothalamic-pituitary adrenal axis function. The level of significance was defined at *α* = 0.05 Lower symptoms of depression post-intervention (*p* = 0.002).
Menengi et al., 2022. Turkey. [19]	Effectiveness of motor-cognitive dual-task exercise via telerehabilitation in Alzheimer’s disease: An online pilot randomized controlled study.	Pilot RCT.	20 participants.	Motor–cognitive dual-task exercise treatment.	Supervised exercise programs.	Reduced anxiety and depressive symptoms, and improved cognition, mobility, and well-being.
Lee et al., 2023. Australia. [20]	A Scalable Program for Improving Physical Activity in Older People with Dementia Including Culturally and Linguistically Diverse (CALD) Groups Who Receive Home Support: A Feasibility Study.	Prospective observational study.	38 participants.	Home exercise program that included knee and ankle strengthening exercises, balance exercises, and walking exercises.	Supervised exercise programs.	Improved physical function, falls efficacy, overall well-being.
Edwards et al., 2013. Australia. [21]	An evaluation of a therapeutic garden’s influence on the quality of life of aged care residents with dementia.	Longitudinal study.	34 participants.	Gardening as a physical activity.	Recreational activities as a form of exercise.	The mean depression and agitation scores decreased by almost half. The mean quality of life score increased (level of significance: *p* < 0.0001).
Levinger et al., 2023. Australia. [22]	Exercise interveNtion outdoor proJect in the cOmmunitY-results from the ENJOY program for independence in dementia: a feasibility pilot randomised controlled trial.	Pilot RCT.	16 participants.	Structured supervised physical activity program that included functional and balance exercises.	Supervised exercise programs.	Improved level of engagement, and mood.
Inskip et al., 2022. Australia. [23]	Promoting independence in Lewy body dementia through exercise: the PRIDE study.	Non-randomised, non-blinded, crossover pilot trial.	9 participants.	High-intensity, progressive exercise training.	Supervised exercise programs.	Improved functional independence, cognition, physical function, and strength, which in turn improved patients’ well-being.
Ellis et al., 2017. Australia. [24]	Laughter yoga activities for older people living in residential aged care homes: A feasibility study.	Prospective observational study.	28 participants.	Laughter yoga program.	Recreational activities as a form of exercise.	Lower mean negative mood score. Measurable improvement in happiness scores (*p* = 0.001).
Telenius et al., 2015. Norway. [25]	Long-term effects of a 12 weeks high-intensity functional exercise program on physical function and mental health in nursing home residents with dementia: a single blinded randomized controlled trial.	Single blinded, RCT.	170 participants.	Intensive strengthening and balance exercises.	Supervised exercise programs.	The level of apathy (which is linked to depression, more rapid cognitive and functional decline, and elevated mortality) was lower in the exercise group compared to the control group (*p* = 0.048).
Hill et al., 2009. Australia. [26]	Effectiveness of balance training exercise in people with mild to moderate severity Alzheimer’s disease: Randomized trial.	RCT.	214 participants.	Home-based balance exercise.	Unsupervised exercise programs.	Reduced falls and fall injuries.
Cancela et al., 2016. Australia. [27]	Effects of a long-term aerobic exercise intervention on institutionalized patients with dementia.	RCT.	51 participants.	Structured exercise program	Supervised exercise programs.	Significant improvement in neuropsychiatric symptoms (*p* = 0.020), memory function (*p* = 0.028) and functional mobility (*p* = 0.043) among participants who exercised.
Sondell et al., 2019. Sweden. [28]	Is the Effect of a High-Intensity Functional Exercise Program on Functional Balance Influenced by Applicability and Motivation among Older People with Dementia in Nursing Homes?	RCT.	81 participants.	A high-intensity functional exercise program.	Supervised exercise programs.	No improvement in mental health or well-being.
Neville et al., 2014. Australia. [29]	Exploring the effect of aquatic exercise on behavior and psychological well-being in people with moderate to severe dementia: A pilot study of the Watermemories Swimming Club.	Prospective observational study.	24 participants.	Aquatic exercises.	Recreational activities as a form of exercise.	Decreased behavioural and psychological symptoms, and improved psychological well-being.
Toots et al., 2016. Sweden. [30]	Effects of a High-Intensity Functional Exercise Program on Dependence in Activities of Daily Living and Balance in Older Adults with Dementia.	Cluster-RCT.	186 participants.	High-intensity functional exercise program.	Supervised exercise programs.	No improvement in mental health.
Stevens et al., 2006. Australia. [31]	A randomised controlled trial testing the impact of exercise on cognitive symptoms and disability of residents with dementia.	RCT.	75 participants.	Exercise program.	Unsupervised exercise programs.	Increased function and independence. Improvement in self-help skills. Level of significance: *p* = 0.019.
Lopez-Bueno et al., 2023. USA. [32]	Moderate and vigorous leisure time physical activity in older adults and Alzheimer’s disease-related mortality in the USA: a dose-response, population-based study.	Population-based study.	91,298 participants.	Moderate-to-vigorous leisure time physical activity.	Unsupervised exercise programs.	Improved well-being and reduced mortality.
Telenius et al., 2015. Norway. [33]	Effect of a high-intensity exercise program on physical function and mental health in nursing home residents with dementia: an assessor blinded randomized controlled trial.	Assessor blinded, RCT.	170 participants.	Strengthening and balance exercises.	Supervised exercise programs.	The level of apathy (which is linked to depression, more rapid cognitive and functional decline, and elevated mortality) was lower in the exercise group compared to the control group.
Suttanon et al., 2013. Australia. [34]	Feasibility, safety and preliminary evidence of the effectiveness of a home-based exercise program for older people with Alzheimer’s disease: a pilot randomized controlled trial.	RCT.	40 participants.	An exercise program supervised by a physiotherapist. Program included balance and strengthening exercises and walking exercise.	Supervised exercise programs.	Increased balance and performance, reduced fall risk, and reduced behavioural and psychological symptoms (*p* < 0.05).
Karamacoska et al. 2023. Australia. [35]	A systematic review of the health effects of yoga for people with mild cognitive impairment and dementia.	Systematic review.	10 articles (total 421 participants).	A yoga program.	Recreational activities as a form of exercise.	Improved cognition and mood.
Vreugdenhi et al., 2012. Australia. [36]	A community-based exercise program to improve functional ability in people with Alzheimer’s disease: a randomized controlled trial.	RCT.	40 participants.	Outdoor exercises including walking.	Unsupervised exercise programs.	Improved cognitive function, physical function, and independence (*p* = 0.001).
Bostrom et al., 2016. Swededn. [37]	Effects of a high-intensity functional exercise program on depressive symptoms among people with dementia in residential care: a randomized controlled trial.	RCT.	186 participants.	A high-intensity functional exercise program.	Supervised exercise programs.	A 4-month high-intensity functional exercise program has no superior effect on depressive symptoms relative to a control activity.
Wesson et al., 2013. Australia. [38]	A feasibility study and pilot randomized trial of a tailored prevention program to reduce falls in older people with mild dementia.	RCT.	38 participants.	Balance- and strength-enhancing exercises.	Supervised exercise programs	No psychological difference between the control and the intervention group. Fewer falls in the control group with 95% CI. All participants demonstrated cognitive impairment in the mild range.
Ho et al., 2020. Hong Kong. [39]	Psychophysiological Effects of Dance Movement Therapy and Physical Exercise on Older Adults With Mild Dementia: A Randomized Controlled Trial.	RCT.	204 participants.	Dance movement therapy.	Supervised exercise programs.	A significant decrease in depression, loneliness, and negative mood (d = 0.33–0.42, *p* < 0.05), and improvement in daily functioning (d = 0.40, *p* < 0.01) and diurnal cortisol slope (d = 0.30, *p* < 0.01).
Brett et al., 2016. Australia. [40]	Effects of Physical Exercise on Health and Well-Being of Individuals Living with a Dementia in Nursing Homes: A Systematic Review.	Systematic review.	12 studies (*n* = 901).	Physical exercises including walking, movements with music, and movement and hand exercises.	Supervised exercise programs.	Most trials reported significant positive effects of physical exercise on cognition, agitation, mood, and well-being.
Middleton et al., 2018. USA. [41]	The Mental Activity and eXercise (MAX) trial: Effects on physical function and quality of life among older adults with cognitive complaints.	RCT.	126 participants.	Aerobic exercise and mental activity training.	Supervised exercise programs and recreational mental activities.	There was no improvement in mental health or quality of life.

All included articles were published from 2005 to 2023. They all used quantitative methodologies. Studies were conducted in high-income countries. The top two countries where research was conducted were Australia and Sweden. The remaining studies were spread across a few countries: United States of America (USA), Norway, Hong Kong, and Turkey. The most common intervention was supervised exercise programs. The majority of physical activity programs were structured exercises such as strength, balance, functional and resistance training, and walking. The study samples were from community settings, residential care facilities, and hospitals.

**Figure 2:**
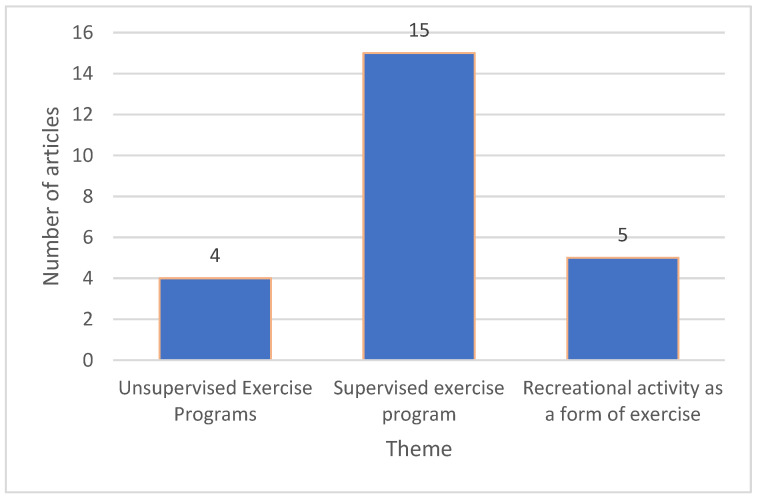
Study themes based on the type of exercise in reviewed studies.

A narrative account was prepared from the included studies to determine the impact of physical activity on the mental health and well-being of dementia patients. The data were synthesised thematically into three main themes (Figure 2):

### 3.2. Unsupervised Exercise Programs

Four studies investigated the impact of unsupervised exercise programs on dementia patients’ mental health and well-being [26,31,32,36]. Studies were conducted in Australia and USA. The studies included three randomised controlled trials [26,31,36] and one population-based study [32]. Participant numbers varied from 91,298 participants to 40 participants. The studies used various types of physical activity including home-based balance exercises, outdoor exercises, and moderate-to-vigorous leisure time physical activity. All studies showed that physical activity improves independence and physical function, which improves patients’ well-being [26,31,32,36]. 

### 3.3. Supervised Exercise Program

Fifteen studies used tailored and supervised exercise programs to explore the impact of physical activity on the mental health and well-being of dementia patients [19,20,22,23,25,27,28,30,33,34,37,38,39,40,41]. Studies were conducted in Norway, Australia, Turkey, Sweden, and Hong Kong and included twelve randomised controlled trials [19,22,25,27,28,30,33,34,37,38,39,41], one non-randomised, non-blinded, crossover pilot trial [23], one prospective observational study [20], and one systematic review (which included 12 studies) [40]. Participant numbers varied from 901 participants to 9 participants. The studies used various types of physical activity including high-intensity, progressive exercise training, strengthening and balance exercises, high-intensity functional exercise programs, dance movements, walking, and aerobic exercises. Most studies showed that supervised exercise programs may reduce anxiety and depressive symptoms, decrease the level of apathy (which is linked to depression, more rapid cognitive and functional decline, and elevated mortality), improve mood, and improve overall well-being [19,20,22,23,25,27,33,34,39,40]. However, a few studies showed that physical activity has no impact on the mental health and well-being of dementia patients [28,30,37,38,41].

### 3.4. Recreational Activity as a Form of Exercise

Five publications used various recreational activities as a form of exercise, to improve the mental health and well-being of dementia patients [18,21,24,29,35]. Studies were conducted in Australia and included an exploratory study [18], a longitudinal study [21], two prospective observational studies [24,29], and one systematic review [35]. Participant numbers varied from 421 participants to 10 participants. The studies used various types of recreational activities including art gallery outings, gardening, laughter yoga, and swimming. These studies showed that physical activity significantly decreases depression and agitation symptoms, improves mood, and the overall well-being of patients with dementia.

## 4. Discussion

This systematic scoping review showed that regular physical activity by way of supervised exercise programs, unsupervised exercise programs, and recreational activities, significantly improves the mental health and well-being of patients with dementia. Only a handful of studies showed that supervised exercise programs may not have an impact on the mental health and well-being of dementia patients. It is possible that these studies did not yield a positive result due to involving high-intensity exercise programs which may have been more strenuous than participants’ ability, which in turn may have led to poor adherence, given the type of participants. A recent study by Rivera-Torres et al. showed that factors that play a significant role in older adults’ participation and adherence to physical activity programs include the level of physical activity, health status, physical ability, cognitive ability, and the existence of depressive symptoms [42]. 

The systematic scoping review found strong evidence of the positive impact of physical activity on dementia patients’ mental health and well-being, with the majority showing positive results. This finding concurs with the findings of other studies which showed that regular physical activity may decrease the rate of depression, improve health status, reduce the rate of falls, improve mobility, and lower functional dependence among older adults, which in turn improves older adults’ well-being [43,44,45,46].

This systematic scoping review found a significant decrease in cognitive impairment among older adults with dementia who engage in regular physical activity. Cognitive functioning is strongly associated with well-being, with growing evidence suggesting that physical activity has tremendous positive effects on the cognitive functioning of those with dementia [47]. The review also demonstrated that individualised interventions that included occupational therapy, physiotherapy, home visits, and follow-up phone calls may significantly improve dementia patients’ cognitive function [48].

Further, the review showed that regular physical activity may lower falls among dementia patients. This in turn may safeguard their mental health and well-being. It is well-established that exercise can contribute to the body’s ability to balance itself during movement, especially during walking or when attempting to get up [49]. A systematic review by Thomas et al., found that elderly participants who followed regular exercise were more likely to be protected against falls [50]. The above study went on to recommend the promotion of physical activity for the aging population. 

This systematic scoping review showed strong evidence that physical activity may have a positive impact on dementia patients’ mental health and well-being. It is well-established that physical activity may improve dementia patients’ mental health and well-being [51]. Due to the multifaceted nature of physical activity in the various studies, it is difficult to pinpoint what may have been the most effective type of physical activity [38]. Furthermore, the impact of additional intervention components such as socialisation, rekindled positive memories, fun, and relaxation are yet to be explored through controlled studies. Nevertheless, the association between physical activity and improved well-being and mental health has been well reported previously [52,53]. Moreover, an earlier systematic review (2010) by Aarsland and colleagues also concluded that regular physical activity may improve the psychological well-being of elderly patients with dementia [54]. 

Although the summary of evidence provided in this systematic scoping review has added strength to the already existing evidence of the positive impact of physical activity on dementia patients’ mental health and well-being, the above analysis covered a relatively small number of studies pertinent to the research question. While all efforts were made to capture any study within the timeframe of the review, some studies may have been missed by chance, which may have potentially impacted the overall outcome. Moreover, the systematic scoping review was limited to studies conducted in high-income countries and therefore, findings may not necessarily apply to dementia patients from poor-income countries. However, the evidence and the effect of exercise on health and well-being may relate to all, regardless of their wealth status. Finally, the inclusion of two systematic reviews in the summary of evidence in this scoping review may have potentially impacted the methodological quality of this review as the systematic reviews collectively included a handful of studies conducted earlier than 2013. 

## 5. Conclusions and Recommendations

This systematic scoping review explored the impact of physical activity on the mental health and well-being of dementia patients in high-income countries. One of the most evident findings was the decrease in cognitive impairment and falls when dementia patients engage in regular physical activity. Such positive outcomes in turn may contribute to the improvement of overall well-being. Moreover, lower depression rates were also recorded when dementia patients engage in regular physical activity. Overall, there was significant evidence of the positive impact of physical activity programs on the mental health and well-being of dementia patients. 

Health policymakers and healthcare providers are highly encouraged to incorporate regular physical activity into the care plan of dementia patients. The regulation and formalisation of these care plans will not only protect dementia patients against complications including falls, cognitive impairment, and poor mental health, but it will also assist in reducing the risk of further chronic illness and/or lowering the impact of any pre-existing chronic disorders. Rigorous studies with large sample sizes may be needed to confirm some of the findings in this systematic scoping review.

## References

[1] World Health Organization (2010). Global Recommendations on Physical Activity for Health.

[2] Dementia. https://www.who.int/news-room/fact-sheets/detail/dementia.

[3] (2022). Welfare AIoHa: Dementia in Australia.

[4] Dementia Australia. Physical Exercise and Dementia, 2015.

[5] Brown L., Hansnata E., La H. Economic Cost of Dementia in Australia 2016–2056: Report Prepared for Alzheimer’s Australia; Australia, 2017. https://researchprofiles.canberra.edu.au/en/publications/economic-cost-of-dementia-in-australia-2016-2056-report-prepared-.

[6] Miskovski K., Alzheimer’s Australia NSW (2014). The Benefits of Physical Activity and Exercise for People Living with Dementia.

[7] Physical Activity. https://www.who.int/health-topics/physical-activity#tab=tab_2.

[8] Larson E.B., Wang L., Bowen J.D., McCormick W.C., Teri L., Crane P., Kukull W. (2006). Exercise is associated with reduced risk for incident dementia among persons 65 years of age and older. Ann. Intern. Med..

[9] Galán-Arroyo C., Pereira-Payo D., Hernández-Mocholí M., Merellano-Navarro E., Pérez-Gómez J., Rojo-Ramos J., Adsuar J.C. (2022). Depression and Exercise in Older Adults: Exercise Looks after You Program, User Profile. Healthcare.

[10] Groot C., Hooghiemstra A.M., Raijmakers P.G., van Berckel B.N., Scheltens P., Scherder E.J., van der Flier W.M., Ossenkoppele R. (2016). The effect of physical activity on cognitive function in patients with dementia: A meta-analysis of randomized control trials. Ageing Res. Rev..

[11] Heyn P., Abreu B.C., Ottenbacher K.J. (2004). The effects of exercise training on elderly persons with cognitive impairment and dementia: A meta-analysis. Arch. Phys. Med. Rehabil..

[12] Hernandez S.S., Coelho F.G., Gobbi S., Stella F. (2010). Effects of physical activity on cognitive functions, balance and risk of falls in elderly patients with Alzheimer’s dementia. Rev. Bras. Fisioter..

[13] Peters M., Godfrey C., Khalil H., McInerney P., Soares C., Parker D. (2017). 2017 Guidance for the Conduct of JBI Scoping Reviews. https://www.researchgate.net/publication/319713049_2017_Guidance_for_the_Conduct_of_JBI_Scoping_Reviews..

[14] Aromataris E., Munn Z. (2020). JBI Manual for Evidence Synthesis. https://jbi-global-wiki.refined.site/space/MANUAL.

[15] Moher D., Liberati A., Tetzlaff J., Altman D.G. (2009). Preferred reporting items for systematic reviews and meta-analyses: The PRISMA statement. J. Clin. Epidemiol..

[16] Ewald H., Klerings I., Wagner G., Heise T.L., Stratil J.M., Lhachimi S.K., Hemkens L.G., Gartlehner G., Armijo-Olivo S., Nussbaumer-Streit B. (2022). Searching two or more databases decreased the risk of missing relevant studies: A metaresearch study. J. Clin. Epidemiol..

[17] Braun V., Clarke V., Cooper H., Camic P.M., Long D.L., Panter A.T., Rindskopf D., Sher K.J. (2012). Thematic analysis. APA Handbook of Research Methods in Psychology, Vol. 2. Research Designs: Quantitative, Qualitative, Neuropsychological, and Biological.

[18] D’Cunha N.M., McKune A.J., Isbel S., Kellett J., Georgousopoulou E.N., Naumovski N. (2019). Psychophysiological Responses in People Living with Dementia after an Art Gallery Intervention: An Exploratory Study. J. Alzheimers Dis..

[19] Menengi Ç.K.N., Yeldan İ., Çınar N., Şahiner T. (2022). Effectiveness of motor-cognitive dual-task exercise via telerehabilitation in Alzheimer’s disease: An online pilot randomized controlled study. Clin. Neurol. Neurosurg..

[20] Lee D.A., Haines T.P., Callisaya M.L., Hill K.D. (2023). A Scalable Program for Improving Physical Activity in Older People with Dementia Including Culturally and Linguistically Diverse (CALD) Groups Who Receive Home Support: A Feasibility Study. Int. J. Environ. Res. Public Health.

[21] Edwards C.A., McDonnell C., Merl H. (2013). An evaluation of a therapeutic garden’s influence on the quality of life of aged care residents with dementia. Dementia.

[22] Levinger P., Goh A.M.Y., Dunn J., Katite J., Paudel R., Onofrio A., Batchelor F., Panisset M.G., Hill K.D. (2023). Exercise interveNtion outdoor proJect in the cOmmunitY—Results from the ENJOY program for independence in dementia: A feasibility pilot randomised controlled trial. BMC Geriatr..

[23] Inskip M.J., Mavros Y., Sachdev P.S., Hausdorff J.M., Hillel I., Singh M.A.F. (2022). Promoting independence in Lewy body dementia through exercise: The PRIDE study. BMC Geriatr..

[24] Ellis J.M., Ben-Moshe R., Teshuva K. (2017). Laughter yoga activities for older people living in residential aged care homes: A feasibility study. Australas. J. Ageing.

[25] Telenius E.W., Engedal K., Bergland A. (2015). Long-term effects of a 12 weeks high-intensity functional exercise program on physical function and mental health in nursing home residents with dementia: A single blinded randomized controlled trial. BMC Geriatr..

[26] Hill K.D., LoGiudice D., Lautenschlager N.T., Said C.M., Dodd K.J., Suttanon P. (2009). Effectiveness of balance training exercise in people with mild to moderate severity Alzheimer’s disease: Protocol for a randomised trial. BMC Geriatr..

[27] Cancela J.M., Ayán C., Varela S., Seijo M. (2016). Effects of a long-term aerobic exercise intervention on institutionalized patients with dementia. J. Sci. Med. Sport.

[28] Sondell A., Littbrand H., Holmberg H., Lindelof N., Rosendahl E. (2019). Is the Effect of a High-Intensity Functional Exercise Program on Functional Balance Influenced by Applicability and Motivation among Older People with Dementia in Nursing Homes?. J. Nutr. Health Aging.

[29] Neville C., Henwood T., Beattie E., Fielding E. (2014). Exploring the effect of aquatic exercise on behaviour and psychological well-being in people with moderate to severe dementia: A pilot study of the Watermemories Swimming Club. Australas. J. Ageing.

[30] Toots A., Littbrand H., Lindelof N., Wiklund R., Holmberg H., Nordstrom P., Lundin-Olsson L., Gustafson Y., Rosendahl E. (2016). Effects of a High-Intensity Functional Exercise Program on Dependence in Activities of Daily Living and Balance in Older Adults with Dementia. J. Am. Geriatr. Soc..

[31] Stevens J., Killeen M. (2006). A randomised controlled trial testing the impact of exercise on cognitive symptoms and disability of residents with dementia. Contemp. Nurse.

[32] Lopez-Bueno R., Yang L., Stamatakis E., Del Pozo Cruz B. (2023). Moderate and vigorous leisure time physical activity in older adults and Alzheimer’s disease-related mortality in the USA: A dose-response, population-based study. Lancet Healthy Longev..

[33] Telenius E.W., Engedal K., Bergland A. (2015). Effect of a high-intensity exercise program on physical function and mental health in nursing home residents with dementia: An assessor blinded randomized controlled trial. PLoS ONE.

[34] Suttanon P., Hill K.D., Said C.M., Williams S.B., Byrne K.N., Logiudice D., Lautenschlager N.T., Dodd K.J. (2013). Feasibility, safety and preliminary evidence of the effectiveness of a home-based exercise programme for older people with Alzheimer’s disease: A pilot randomized controlled trial. Clin. Rehabil..

[35] Karamacoska D., Tan T., Mathersul D.C., Sabag A., de Manincor M., Chang D., Steiner-Lim G.Z. (2023). A systematic review of the health effects of yoga for people with mild cognitive impairment and dementia. BMC Geriatr..

[36] Vreugdenhil A., Cannell J., Davies A., Razay G. (2012). A community-based exercise programme to improve functional ability in people with Alzheimer’s disease: A randomized controlled trial. Scand. J. Caring Sci..

[37] Bostrom G., Conradsson M., Hornsten C., Rosendahl E., Lindelof N., Holmberg H., Nordstrom P., Gustafson Y., Littbrand H. (2016). Effects of a high-intensity functional exercise program on depressive symptoms among people with dementia in residential care: A randomized controlled trial. Int. J. Geriatr. Psychiatry.

[38] Wesson J., Clemson L., Brodaty H., Lord S., Taylor M., Gitlin L., Close J. (2013). A feasibility study and pilot randomised trial of a tailored prevention program to reduce falls in older people with mild dementia. BMC Geriatr..

[39] Ho R.T.H., Fong T.C.T., Chan W.C., Kwan J.S.K., Chiu P.K.C., Yau J.C.Y., Lam L.C.W. (2020). Psychophysiological Effects of Dance Movement Therapy and Physical Exercise on Older Adults With Mild Dementia: A Randomized Controlled Trial. J. Gerontol. B Psychol. Sci. Soc. Sci..

[40] Brett L., Traynor V., Stapley P. (2016). Effects of Physical Exercise on Health and Well-Being of Individuals Living with a Dementia in Nursing Homes: A Systematic Review. J. Am. Med. Dir. Assoc..

[41] Middleton L.E., Ventura M.I., Santos-Modesitt W., Poelke G., Yaffe K., Barnes D.E. (2018). The Mental Activity and eXercise (MAX) trial: Effects on physical function and quality of life among older adults with cognitive complaints. Contemp. Clin. Trials.

[42] Rivera-Torres S., Fahey T.D., Rivera M.A. (2019). Adherence to Exercise Programs in Older Adults: Informative Report. Gerontol. Geriatr. Med..

[43] Ajmiri M.Y., Bahir H. (2023). Impact of physical exercise on the mental health of the elderly. Sustain. Sports Sci. J..

[44] Callow D.D., Arnold-Nedimala N.A., Jordan L.S., Pena G.S., Won J., Woodard J.L., Smith J.C. (2020). The Mental Health Benefits of Physical Activity in Older Adults Survive the COVID-19 Pandemic. Am. J. Geriatr. Psychiatry.

[45] Agbangla N.F., Séba M.-P., Bunlon F., Toulotte C., Fraser S.A. (2023). Effects of Physical Activity on Physical and Mental Health of Older Adults Living in Care Settings: A Systematic Review of Meta-Analyses. Int. J. Environ. Res. Public Health.

[46] Yao L., Fang H., Leng W., Li J., Chang J. (2021). Effect of Aerobic Exercise on Mental Health in Older Adults: A Meta-Analysis of Randomized Controlled Trials. Front. Psychiatry.

[47] Llewellyn D.J., Lang I.A., Langa K.M., Huppert F.A. (2008). Cognitive function and psychological well-being: Findings from a population-based cohort. Age Ageing.

[48] So M., Foxe D., Kumfor F., Murray C., Hsieh S., Savage G., Ahmed R.M., Burrell J.R., Hodges J.R., Irish M. (2018). Addenbrooke’s Cognitive Examination III: Psychometric Characteristics and Relations to Functional Ability in Dementia. J. Int. Neuropsychol. Soc..

[49] Granacher U., Gollhofer A., Hortobágyi T., Kressig R.W., Muehlbauer T. (2013). The importance of trunk muscle strength for balance, functional performance, and fall prevention in seniors: A systematic review. Sports Med..

[50] Thomas E., Battaglia G., Patti A., Brusa J., Leonardi V., Palma A., Bellafiore M. (2019). Physical activity programs for balance and fall prevention in elderly: A systematic review. Medicine.

[51] Anderiesen H., Scherder E.J., Goossens R.H., Sonneveld M.H. (2014). A systematic review–physical activity in dementia: The influence of the nursing home environment. Appl. Ergon..

[52] Colcombe S., Kramer A.F. (2003). Fitness effects on the cognitive function of older adults: A meta-analytic study. Psychol. Sci..

[53] Blondell S.J., Hammersley-Mather R., Veerman J.L. (2014). Does physical activity prevent cognitive decline and dementia? A systematic review and meta-analysis of longitudinal studies. BMC Public Health.

[54] Aarsland D., Sardahaee F.S., Anderssen S., Ballard C. (2010). Is physical activity a potential preventive factor for vascular dementia? A systematic review. Aging Ment. Health.

